# Supplementary material for: Are carbon emissions trading and green financial instruments synergistic? -Comprehensive quantitative research based on content analysis
Source: PLoS One. 2024 Mar 7;19(3):e0298601. doi: 10.1371/journal.pone.0298601 (PMC10919601; doi:10.1371/journal.pone.0298601)
Supplement: S1 Data — (ZIP) [file pone.0298601.s001.zip › Data/地级市Z_F工作报告.zip!/╕╩╦α/╢¿╬≈╩╨/└┤╫╘╣1⁄2╓┌║┼í╛┬φ┐╦╩2╛▌═°í┐_2017.docx]

各位代表：

　　现在，我代表市人民政府，向大会报告政府工作，请予审议，并请各位政协委员和其他列席人员提出意见。

过去五年工作回顾

　　市三届人大一次会议以来，在省委、省政府和市委的坚强领导下，在市人大及其常委会、市政协的监督支持下，市政府认真贯彻党的十八大和十八届三中、四中、五中、六中全会精神，深入学习贯彻习近平总书记系列重要讲话精神，主动适应经济发展新常态，有效应对各种风险和挑战，完成了任期主要目标任务。

　　（一）千方百计扩投资稳增长，经济实力持续提升。抢抓政策叠加机遇，致力扩大投资规模，促进发展速度和效益同步提升，主要经济指标增速居全省中上水平。预计到2016年底，完成生产总值321亿元，是2011年的1.72倍，年均增长10%。人均生产总值突破万元大关，达到11541元。实施500万元以上项目7475个，其中亿元以上项目852个，累计完成固定资产投资2600亿元，是前五年的3.5倍，年均增长17.2%。工业增加值达到35亿元，是2011年的1.1倍，年均增长13.6%。社会消费品零售总额达到117亿元，是2011年的1.84倍，年均增长13%。大口径财政收入47.24亿元，是2011年的2.7倍；一般公共预算收入25亿元、支出206亿元，分别是2011年的2.5倍和1.7倍。金融机构存贷款余额成倍增长，分别达到808亿元和690亿元。城乡居民人均可支配收入分别达到20700元和6464元，比2011年增加8410元和3002元，年均增速分别达到11%和13.3%。三次产业比由28∶28∶44调整为24∶21∶55。

　　（二）精准施策真扶贫扶真贫，脱贫攻坚扎实推进。坚持把脱贫攻坚作为“一号工程”，建立完善机构整合、力量充实、联系包抓、投入保障等机制，全面落实“1+16+5”精准脱贫政策措施，严格实行“853”挂图作业，全市财政累计投入综合扶贫资金615亿元，减少贫困人口72万人。大力推行“四跟进”产业扶贫模式，实施总投资112亿元的产业项目79个，引导908户企业与849个贫困村、5800多个合作社建立结对帮扶关系，带动30万户群众增收致富，农民人均产业收入占可支配收入的60%以上。加快发展电子商务、光伏、旅游等富民多元产业，建成7个县级电子商务服务中心、89个乡级服务站和1070个村级服务点，岷县、渭源县被列入国家电子商务进农村综合示范县，通渭、临洮600户分布式光伏扶贫试点项目全面完成，7县区全部列入全省光伏扶贫试点县，140个村纳入国家乡村旅游扶贫工程实施范围。积极整合各类培训资源，推行“两重点、五统一”精准培训模式，累计培训贫困劳动力30万人次。加快贫困村基础设施建设，提升公共服务水平，农村自来水普及率提高到90%，改造农村危房10.48万户，易地扶贫搬迁16.4万人，建成农村道路7517公里，行政村水泥（沥青）路、标准化卫生室、农家书屋、贫困自然村动力电和1500人以上行政村幼儿园实现全覆盖。强化金融保障，发放精准扶贫专项贷款65.95亿元、互助资金担保增信贷款4亿元，扶持15万户贫困群众发展增收致富产业。“三西”扶贫开发现场会、首届中国扶贫论坛、全省基层党建助推精准扶贫现场会等重大活动在我市举办，定西脱贫攻坚的探索与实践产生广泛影响。

　　（三）坚持不懈抓转型促升级，产业发展势头良好。投入财政资金10.78亿元，扶持优势特色产业全产业链发展。围绕打造“中国药都”，推进国家中医药产业发展综合试验区先行先试工作，中药材种植面积稳定在150万亩左右，加工和仓储能力分别达到35万吨和85万吨，新建中医药精深加工项目91个，“岷归”“扶正”荣获中国驰名商标，陇西县创建为全国中药材种植产业知名品牌示范区。围绕打造“中国薯都”，推进马铃薯主食化战略先行试验示范区建设，成立主食化产业开发联盟，马铃薯种植面积稳定在300万亩左右，加工和贮藏能力分别达到60万吨和350万吨，脱毒原原种生产能力达到10亿粒以上，建成薯香园主食化产品开发等项目31个，国家级马铃薯专业批发市场一期工程建成投用，“定西马铃薯”荣获中国驰名商标，“陇上绿莹”牌马铃薯及其制品被认定为全国首家马铃薯生态原产地保护产品，安定区创建为全国马铃薯产业知名品牌示范区。围绕打造“中国西部草都”，成立牧草产业科技创新战略联盟，草产量突破700万吨，加工能力达到500万吨，畜禽饲养总量和肉蛋奶总产量分别达到2600万头只和33万吨，规模化养殖比重提高到46%，种养加销一体化进程明显加快。蔬菜种植面积达到70万亩，总产量270万吨，建成林果基地30万亩。深入推进“工业强市”战略，实施千万元以上工业项目812个，完成投资705亿元。新增规模以上企业116户，累计达到160户，完成增加值29.5亿元、年均增长14.2%。大力推进开发区和工业集中区建设，不断优化产业布局，提升承载能力，建成区面积扩大到42.6平方公里，引进入驻企业1202户，完成投资500亿元。组建省市级工程技术研究中心168个，培育高新技术企业22家。中天羊业、巨鹏食品、甘肃金海成功登陆“新三板”，84家企业在区域性股权交易市场挂牌，直接融资1.98亿元。主导设立甘肃生物医药产业创业投资基金，投入资金2.1亿元，扶持10户创新型企业发展壮大。现代服务业加快发展，通渭书画产业园、陇西古莱坞文化旅游创意园等项目建成运营，渭河源大景区创建为国家4A级旅游景区并被列入全省20个大景区，文化产业增加值年均增长24%，旅游综合收入年均增长20%。引进建设西部国际汽配城等亿元以上现代商贸物流项目20个，建成产地特色农产品交易市场20个，城乡便民服务市场100个。邮政快递、健康养老等新兴业态快速兴起，服务业增加值年均增长11%。

　　（四）全力以赴补短板夯基础，发展后劲明显增强。加快推进新型城镇化进程，完成城镇建设投资542亿元，是前五年的2倍，市县建成区面积扩大到93.7平方公里，21万农业人口进城落户，城镇化率达到32.2%，较2011年提高7.8个百分点。市县城区总体规划完成修编，乡镇总体规划编制实现全覆盖。市区新城区8纵12横道路框架基本形成，市行政中心整体搬迁，定西公园、生态园等公共服务项目建成投用，亚行贷款“六路三桥”等项目进展顺利。老城区交通路改造等29个项目全面建成，西关什字等4座过街人行天桥建成投用，正龙路、中华路北段、友谊北路3条丁字路全部打通，路网结构更加优化。各县城区和重点小城镇基础设施不断完善，承载能力明显提升。集中开展城区环境综合整治，完善长效管理机制，着力解决群众反映强烈的物业管理、供热服务、占道经营等突出问题，城市管理水平不断提高。加快实施重大基础设施项目，兰渝铁路岷县至广元段、临渭高速年内通车，宝兰客专、渭武高速加快建设，定临、文殪、马云二级公路建成通行，县区之间实现二级以上公路连接，所有乡镇通等级公路。天水至陇西输气管道投入运行，兰州至定西输气管道基本建成。引洮一期工程建成通水、二期工程有序推进，新增灌溉面积16.1万亩，160多万群众喝上了甘甜的洮河水。渭河源区生态保护与综合治理规划批复实施，定西被列为全国第一批生态文明建设先行示范区。完成造林封育84万亩，新修梯田136.7万亩，新增水土流失治理面积1949平方公里，森林覆盖率、水土流失综合治理程度分别达到13%和53%。实施土地开发整理项目219个，新增耕地7.6万亩。多措并举防治大气污染，PM10治理取得积极进展，市区空气质量优良天数占比保持在81%以上。全面开展河沟渠道污染综合整治，市区东河、西河沿岸污水直排问题初步解决，城镇生活污水处理能力进一步增强，国控断面和饮用水源地水质达标率均为100%。集中开展农村环境连片整治，建成美丽示范村98个、环境整洁村450个，农村人居环境持续改善。

　　（五）凝心聚力抗大灾抓重建，灾区面貌焕然一新。面对突如其来的“5·10”岷县特大冰雹山洪泥石流、“7·22”岷县漳县6.6级地震等重特大自然灾害，全市上下众志成城、共克时艰，干部群众戮力同心、苦干实干，社会各界无私援助、鼎力支持，奋力夺取了抢险救灾全面胜利，圆满完成了灾后恢复重建任务。实施“7·22”岷漳地震灾后重建项目1431个，完成投资119亿元。新建城乡居民住房6.88万户、维修加固7.96万户，66万群众入住新居，住房条件得到历史性改善。1690户7711名受灾群众搬迁到靖远县北湾镇安家落户，生产生活条件彻底改变。重建各类学校183所、乡镇卫生院37个、村卫生室209个，公共服务保障水平显著提升。建成农村公路2039公里，完成国道212、316线改造，实施人饮工程46个，基础设施条件大幅提升。岷县梅川茶埠、漳县大草滩等7个小城镇完成风貌改造，永星等15个重灾村和永光等48个集中安置点全部建成。实施“5·10”岷县特大雹洪泥石流灾后重建项目293个，完成投资22亿元。实施“4·15”临洮4.5级地震灾后重建项目42个，完成投资6500万元。灾害预警和应急救援机制不断完善，防灾减灾能力有效增强，灾区面貌焕然一新。

　　（六）坚定不移抓改革促开放，内生动力有效激发。8项全国改革试点和10项全省改革试点深入推进。陇西农村集体经营性建设用地挂牌入市敲响全省第一槌。综合执法试点在市县乡全面铺开，执法力量集中下沉。组建成立市公共资源交易中心，全市公共资源交易实现全程电子化。市水务投资集团组建运营,水务一体化改革不断深化。不动产统一登记制度改革有力推进，7县区和市本级实现颁发新证全覆盖。在全省率先试行大病保险、分级诊疗和支付方式改革，率先推行“13885”健康促进模式改革。中药材产值保险累计承保31万亩、赔付5000多万元。商事制度改革扎实推进，新增市场主体6万多户，非公经济占比由40%提高到52%。国企改革有序推进，陇西渭水酒业集团完成改制。甘肃银行、兰州银行入驻定西，临洮农商行、定西农商行、金城村镇银行组建运营。深入开展“千亿元大招商”活动，实施招商引资项目669个，到位资金1850亿元，是前五年的7.4倍，年均增长42%，华能集团和山东招金、史丹利、天津亿联、天士力、广东一方、康美等大型企业落户定西、投资兴业。

　　（七）尽心竭力惠民生促和谐，人民福祉不断增进。财政累计投入民生资金476亿元，占财政支出的比重由37%提高到59%。城镇新增就业14.97万人，失业人员再就业3.6万人，高校毕业生就业2.24万人。市县城区新建各类学校22所，改造校舍175.8万平方米，办学条件大幅改善，学校布局更趋合理。学前三年入学率提高到74.7%，九年义务教育巩固率达到90.5%，高中阶段教育毛入学率达到90.05%，高考二本以上上线率达到33.8%，教育教学质量稳步提升。定西师专、定西卫校整体并入甘肃中医药大学，临洮农校与甘肃农业大学、定西职业技术学院与兰州石化职业技术学院合作办学取得实质性进展。优化整合市区医疗卫生资源，市医院、市二院完成搬迁，市医院与兰大二院医疗集团开展深度合作。二孩政策全面落实，我市创建为全国计划生育利益导向示范市。城乡低保、农村五保和孤儿救助标准不断提高，累计发放各类救助资金55亿元。加快科技成果转化步伐，陇西、安定等5县区创建为国家科技进步先进县区。农村广播电视实现全覆盖，市县本级数字广播电视覆盖率达到70%以上。市县“四馆”免费开放，定西剪纸等7个项目入选国家级非遗名录。定西大剧院和扶贫开发纪念馆、药都博物馆、薯都博物馆全面建成投入使用。全民健身活动蓬勃开展，定西体育馆基本建成。深入开展“双拥”活动，安定等6个县区创建为省级双拥模范县区。严格靠实工作责任，食品药品安全监管得到加强，安全生产形势总体平稳。深化“平安定西”和“法治定西”建设，强化社会治安综合治理，“六五”普法获全省考核优秀等次，八类主要案件和非正常上访大幅下降，社会大局保持和谐稳定。民族宗教、外事侨务、地震、粮食、气象、通信等工作以及妇女、儿童、青少年、老年人、残疾人、社会慈善等事业取得新进步。

　　（八）从严从实抓作风转职能，自身建设切实加强。深入开展党的群众路线教育实践活动、“三严三实”专题教育和“两学一做”学习教育，全市政府系统工作作风持续改进。坚持常务会议会前学法制度，推行政府法律专家咨询委员会制度，强化规范性文件审查备案，严格落实重大事项社会稳定风险评估制度，加强行政复议，政府工作法制化水平不断提高。市县两级政府机构改革全面完成，精简机构23个。深化“放管服”改革，累计承接、取消、调整和下放行政审批事项523项、取消前置审批168项，在全省率先建成“三张清单一张网”并实现“网上行权”，积极推行“双随机一公开”监管，政府行政效能有效提升。电子民生平台累计办理民生事项36万件，群众反映问题诉求渠道进一步畅通。严格落实党风廉政建设“3783”主体责任体系和“866”衡量检验标尺，深入开展效能风暴行动和政风行风评议活动。严格执行中央“八项规定”及省委、市委相关规定，精简会议文件，厉行勤俭节约，市级行政机关公务用车制度改革基本完成，“三公”经费年均下降11%。自觉接受市人大常委会工作评议、专题询问，积极配合开展人大工作试点、地方立法、调研视察和执法检查，办理人大代表建议和政协委员提案1500多件，物业管理和河道污染防治两个条例进入立法程序。

　　各位代表！2016年，我们按照市三届人大七次会议安排部署，积极应对经济下行压力，攻坚克难，奋力前行，全市经济社会发展稳中有进、稳中向好，实现了“十三五”良好开局。

　　一是持续推进脱贫攻坚。认真落实市委市政府《关于打赢脱贫攻坚战的实施意见》，投入财政综合扶贫资金167亿元，占总支出的75.9%，预计减少贫困人口14万人，贫困发生率下降到12.3%。整合各类产业扶贫资金1.87亿元，培育农业产业化龙头企业156家、家庭农场229个，通过流转土地、生产资料入股等方式带动贫困群众分红增收。培训贫困劳动力13万人次，输转63.5万人次，实现劳务收入110亿元。市县区财政为33个脱贫难度最大的贫困村每村落实产业发展补助资金300万元。解决了215个贫困村22万人饮水不稳定问题，完成新一轮农网改造，新建改建农村公路1311公里，改造农村危房1.65万户。积极开展抗旱救灾，努力降低灾害损失。

　　二是推动经济稳定增长。实施500万元以上项目2000个，完成投资622亿元。8个省列重大项目和100个市列重点项目完成年度建设任务。争取国家专项基金建设项目63项16.9亿元，撬动社会投资60亿元。储备政府和社会资本合作项目71个，开工建设18个，完成投资9.5亿元。实施招商引资项目355个，到位资金420亿元，同比增长18%。第22届“兰洽会”和首届敦煌文博会签约项目135个，总投资728亿元，已开工91个。实施千万元以上工业项目168个，完成投资115亿元，实施企业技改项目102个，工业经济企稳向好。加快培育消费新亮点，新增规模以上服务业企业12户，完成社会消费品零售总额117亿元，同比增长9%。

　　三是加快供给侧结构性改革。中药材配方颗粒生产前期工作有力推进，成功举办第二届世界黄芪论坛，定西黄芪道地药材产地通过中华中医药学会认证。蓝天淀粉公司等4户全国马铃薯主食化试点企业投入生产，主食化加工能力突破10万吨，研发马铃薯主食产品18类。成功举办草牧业发展高层研讨会，民祥牧草等龙头企业不断发展壮大，7家养殖企业创建为省级标准化养殖示范场。全面落实“三去一降一补”任务，房地产去库存126万平方米，红旗山煤矿列入全省煤炭行业去产能关闭计划，退出煤炭产能15万吨。深入推进“营改增”改革，积极落实“五缓四降四补贴”政策，切实减轻企业负担，减税红利加快释放。

　　四是加强基础设施建设。市区供热、综合管廊专项规划编制完成，陇西县“多规合一”总体规划批复实施。市区建设完成投资51亿元，滨河西路改扩建、城区供热管网改造、新城区临时停车场等24个项目建成投用，渭源街热源厂、定西西站站前广场、农产品交易中心等50个项目加快推进。各县城实施城建项目140个，完成投资69亿元。通渭平襄书画小镇、临洮洮阳马家窑洮砚小镇、陇西首阳中药材小镇列入全省18个特色小镇启动建设。定西民用机场前期工作进展顺利，通渭至定西、定西至临洮、陇西至漳县3条地方高速前期工作基本完成，通渭至定西高速公路试验段开工建设。通榜、巉郭等8条省道建设完成年度任务。实施临洮东部农村集中供水等水利项目38个，完成投资7.7亿元。完成新一轮退耕还林工程15.4万亩，农村“四旁”、城镇面山义务植树2200万株。

　　五是切实保障改善民生。实施“全面改薄”项目563个，改造校舍14万平方米。临洮县义务教育均衡发展通过国家级评估验收。推进乡村教师支持计划，提高工资待遇等措施得到落实。全面推行县级公立医院药品零差率销售，降低患者医疗费用4000万元。实行新农合包干付费，住院报销比例达64%，较上年提高5.8个百分点。探索开展全市农村“两保一孤”人身意外伤害附加重大疾病保险。实施棚户区改造5103户，建成保障性安居工程3066套，发放低收入家庭住房租赁补贴253万元。建成“乡村舞台”490个，48个乡镇综合文化站实现达标。

　　各位代表！过去五年，是我市经济社会发展成效最好、脱贫攻坚力度最大、城乡面貌变化最快、人民群众得实惠最多的时期之一。这些成绩，是在经济下行压力持续加大的严峻形势下取得的，是在重特大自然灾害频发的复杂环境中取得的，是在脱贫攻坚任务异常繁重的现实条件下取得的。这是党中央、国务院亲切关怀的结果，是省委、省政府和市委坚强领导的结果，是市人大及其常委会和市政协监督支持的结果，更是全市广大干部群众艰苦奋斗的结果。在此，我代表市人民政府，向全市各行各业的建设者，向各级人大代表、政协委员，向各人民团体、民主党派和无党派人士，向人民解放军、武警部队和公安民警，向为定西发展作出贡献的老领导、老同志，向所有关心支持定西发展的社会各界人士，表示衷心的感谢和崇高的敬意！

　　各位代表！成绩来之不易，奋斗充满艰辛。五年来的发展实践使我们深刻体会到：要始终坚持发展为重，立足定西经济欠发达的基本市情，紧紧扭住发展第一要务不放松，走好投资拉动、产业支撑、城镇带动的路子，千方百计谋发展，心无旁骛搞建设；要始终坚持民生为本，任何时候把群众的安危冷暖放在心上，倾力解决上学、看病、就业和社会保障等民生问题，全面发展社会各项事业，让人民群众共享改革发展成果；要始终坚持改革创新，深入实施创新驱动战略，全面深化各项改革，扩大对外开放，向改革要出路、以创新求发展、以开放促开发，不断增强发展动力和活力，着力提高发展质量和效益；要始终坚持务实苦干，不断深化市情认识，创新完善发展思路，大力弘扬“三苦”精神，强化责任使命担当，推动各项政策措施落地见效。

　　回顾过去五年的工作，我们也清醒地看到，经济总量小、人均水平低、贫困程度深、产业结构不合理、生态环境脆弱仍然是我市最基本的市情，加快发展的任务十分繁重。全市经济社会发展中还存在不少困难和问题：一是经济下行压力持续加大，投资增长乏力、消费活力不足、资源要素趋紧，推动经济平稳健康发展的任务依然艰巨；二是我市贫困、生态、灾害问题叠加，致贫返贫因素复杂，巩固脱贫成果难度大，脱贫攻坚的任务依然艰巨；三是传统优势特色产业总体处于增收链、价值链低端，战略性新兴产业规模不大，现代服务业发展滞后，产业转型升级的任务依然艰巨；四是城乡基础设施建设欠账较大，城市功能还不完善，统筹城乡发展的任务依然艰巨；五是社会事业发展仍然滞后，关系群众切身利益的就业创业、教育卫生、社会保障、食品药品、安全生产等方面还存在薄弱环节，保障改善民生的任务依然艰巨；六是政府系统少数工作人员仍存在庸政、懒政、怠政现象，转变政府职能、提高行政效能的任务依然艰巨。对这些问题，我们将在新的发展阶段认真研究解决。

今后五年的总体要求和目标任务

　　今后五年，是我市打赢脱贫攻坚战、全面建成小康社会的决胜期。随着国家加快脱贫攻坚、新一轮西部大开发、新型城镇化、创新驱动、供给侧结构性改革等重大战略举措的深入实施，我省精准脱贫“1+17”专项方案、贯彻新发展理念“1+19”政策体系、丝绸之路经济带“13685”发展战略、“371”优势产业链培育发展行动等一系列政策措施的深入推进，必将为我市加快脱贫攻坚、培育壮大产业、夯实发展基础、补足补齐短板带来难得机遇。刚刚闭幕的市第四次党代会，提出了“建设幸福美好新定西”的奋斗目标，绘制了今后五年发展的宏伟蓝图。我们要坚定信心、抢抓机遇、真抓实干，奋力夺取全面建成小康社会的伟大胜利。

　　根据市第四次党代会精神，政府工作的总体要求是：坚持以邓小平理论、“三个代表”重要思想和科学发展观为指导，深入学习贯彻习近平总书记系列重要讲话精神，紧紧围绕统筹推进“五位一体”总体布局和协调推进“四个全面”战略布局，牢固树立和贯彻落实“五大发展理念”，全力打造中国药都、中国薯都和中国西部草都，深入实施脱贫攻坚、产业升级、城乡建设和生态治理“四大工程”，坚决打赢脱贫攻坚战，努力与全省全国同步全面建成小康社会。

　　经济社会发展的主要预期目标是：生产总值年均增长8%以上，固定资产投资年均增长15%左右，大口径财政收入和一般公共预算收入年均增长10%，城乡居民人均可支配收入年均分别增长8%和12%。

　　围绕上述要求和目标，政府工作突出以下六个方面：

　　（一）决战决胜脱贫攻坚，全面建成共建共享的小康社会。坚持把主要精力放在脱贫攻坚上，集中人力财力物力，坚决打赢脱贫攻坚战，实现2019年全市整体脱贫摘帽。着力强化产业扶贫，加快提升基础设施和公共服务水平，组织实施“七个一批”脱贫工程。大力推行“四跟进”产业扶贫模式，支持贫困村、贫困户因地制宜发展富民多元产业，多渠道增加农民收入，通过发展产业脱贫一批；精准培训贫困劳动力，打造特色劳务品牌，加大劳务输转力度，通过劳务输转脱贫一批；推广“易地扶贫搬迁+”模式，对生态环境恶劣地区的贫困人口实施易地搬迁，从根本上改善生存和发展环境，通过易地搬迁脱贫一批；实施生态治理和保护项目，把有条件的贫困人口就地转化为生态管护人员，通过生态补偿脱贫一批；加快农村薄弱学校改造和贫困村幼儿园建设，全面落实贫困家庭学生教育资助政策，阻止贫困代际传递，通过发展教育脱贫一批；实施健康扶贫工程，健全农村慢性病等预防治疗机制，落实大病保险等医疗救助政策，通过医疗救助脱贫一批；逐年提高农村低保和五保供养标准，持续推行“两保一孤”保险等特困群体扶持政策，实现农村特殊群体养老保险全覆盖，通过社保政策兜底脱贫一批。实施县乡道路改造升级及村组道路建设、农村饮水安全巩固提升和农网供电质量提升工程，全面完成农村危房改造任务，提高贫困群众水电路房保障水平。整合专项资金、涉农资金、社会帮扶资金，确保市县区财政综合扶贫投入稳步增长。加大金融支持力度，提高精准扶贫专项贷款使用效益，实施互助资金担保增信计划，引进保险资金参与脱贫攻坚。深化定点帮扶和东西部扶贫协作，主动与福建省福州市等发达地区对接，重点在产业合作、劳务协作、人才支援、资金支持、合作办学等方面取得实效，积极探索合作共赢的有效机制，最大限度调动社会力量和资源助推脱贫攻坚。完善建档立卡和大数据平台建设，实行贫困人口动态管理，规范退出机制。严格落实“4342”脱贫验收责任体系和精准脱贫问责办法，坚决防止数字脱贫、虚假脱贫。紧密衔接脱贫目标与小康目标，全力补齐人均生产总值等14项弱项指标，加快全面小康实现进程。

　　（二）推动经济转型升级，全面建成支撑有力的小康社会。以供给侧结构性改革为抓手，全力打造中医药、马铃薯、草牧、果蔬4条两百亿元产业链和文化旅游、生物制药、现代物流、新能源4条百亿元产业链，促使一二三产融合发展，推动产业向中高端迈进。

　　加快优势特色产业转型升级。中医药产业要加快推进国家中医药产业发展综合试验区先行先试，力争配方颗粒生产企业达到15家以上。争取将党参、黄芪列入国家新资源食品目录，完成党参、当归国家道地药材产地认证，岷县创建全国当归种植产业知名品牌示范区。全面建成定西中药材商品交易中心和康美甘肃西部中药城，把定西打造成西部最大的中药材集散中心、商贸物流中心和现代中医药产业中心。马铃薯产业要充分发挥主食化开发联盟作用，强化良种繁育和质量检测，加快实施一批主食化精深加工项目，把定西打造成全国脱毒马铃薯良种基地和国家级马铃薯主食产品及产业开发核心区。加快国家级马铃薯批发市场扫尾建设，提高运营管理水平，使其真正成为物流集散、价格形成、信息发布、科技交流、会展贸易中心。草牧产业要依托牧草产业科技创新战略联盟，扶持民祥、天耀和方正等龙头企业做大做强，争取引进大北农、新希望等国内知名草牧企业来我市投资兴业，加快建设西部最大的草产业基地和全省草牧产业大市。果蔬产业要科学高效利用引洮水资源，扩大灌区沿线蔬菜种植规模，种植面积稳定在80万亩左右，因地制宜发展苹果、核桃、梨、大果沙棘、油用牡丹等林果基地56万亩，把定西建成全省高原夏菜和林果生产基地。

　　积极构建新型工业体系。认真实施《<中国制造2025>甘肃行动纲要》和质量强市战略，积极促进制造业与信息技术融合发展，推动有色冶金、新型建材、装备制造等七大主导产业由生产型向市场需求型转变，生产方式向优质高效转变。突出企业主体地位，加强院企、校企合作，组建产业技术创新战略联盟，培育国家级工程技术研究中心1个、省级工程技术研究中心和重点实验室10个以上，高新技术企业发展到30家以上，不断提升企业自主创新能力。完善开发区基础设施和公共服务设施，强化园区土地集约节约利用，提高投资强度，开发区建成区面积扩大到50平方公里以上，力争定西、陇西经济开发区升格为国家级开发区，推动产城融合发展。

　　培育壮大战略性新兴产业。文化旅游业要以建设陇中特色文化大市为目标，推动文化旅游融合发展，全面建成陇西李氏、临洮马家窑、岷县洮砚、通渭书画等文化产业园，通渭县创建全国书画产业知名品牌示范区，文化产业增加值年均增长20%以上。改善提升景区基础设施和服务功能，加快建设旅游景区通用机场和房车露营基地，争取贵清山/遮阳山和渭河源大景区晋升为国家5A级旅游景区，狼渡滩湿地列为国家级湿地公园。精心策划打造精品主题旅游线路，旅游接待人数和旅游收入年均增长20%以上。生物制药业要以提升科技含量为核心，引导扶持一方、扶正、金羚、聚信等生物制药企业与中国农科院、甘肃中医药大学等科研院校深度合作，加强技术研发和联合攻关，推进中药向预防、康复、养生保健等方向延伸，提升产品核心竞争力。现代物流业要抢抓大兰州、关天和成渝经济区发展国际物流的政策机遇，打造陆路进出口货物集散中心。推行“互联网+高效物流”模式，大力发展电子商务，完善城乡快递服务网络，加快岷县、渭源县国家电子商务进农村综合示范县和定西特产电子商务综合体建设，物流业产值占服务业总产值的比重达到20%。新能源产业要重点实施总装机容量170万千瓦的10个风电场项目，加快建设通渭百万千瓦级风电基地。完成7县区光伏扶贫试点项目建设任务，打造全国光伏扶贫示范区。

　　（三）补足补齐发展短板，全面建成基础坚实的小康社会。紧盯国家投资导向和市场需求，深入实施“3341”项目工程、“6873”交通突破行动、“6363”水利保障行动，大力推进“十大支撑工程”建设，加快实施“十三五”规划的695个重点项目，力争固定资产投资突破1000亿元。创新重点领域投融资机制，放宽民间投资市场准入，优化民间投资发展环境，鼓励支持社会资本参与公共服务设施、社会事业、生态环保等领域投资建设。总结推广“多规合一”试点经验，实现市县城区、建制镇控制性详规和乡镇、村庄规划全覆盖。加大规划执行力度，提高规划的权威性和约束力。市区新城区突出生产性和生活性服务设施配套建设，引导人口加快聚集。老城区注重完善道路、管网等市政设施和水、电、气等公共设施，加大棚户区和老旧小区改造力度。深化户籍制度改革，引导农业转移人口市民化，每年新增城镇人口4万人，城镇化率达到40%以上。支持安定积极创建国家级产城融合示范区，加快建设通渭书画城和渭源、漳县、岷县生态园林城市，力争陇西、临洮“撤县建市”。积极创建一批市级特色小镇，争取更多特色小镇进入省级和国家级特色小镇行列。探索推广“大城管”管理模式，提高城镇精细化管理水平。抢抓国家加快中西部铁路建设的机遇，加快推进兰州至天水城际铁路定西段建设，力争环兰州城际铁路定西段、西延铁路岷县段、陇渭铁路开工建设，实现7县区铁路全覆盖。加快高速公路建设，全面建成渭武高速和3条地方高速。大力推进定西民用机场和临洮军民合用机场建设，全面构建现代立体交通网络，实现公路畅通、铁路连通、航路广通目标。加强国省道干线改造和农村公路养护管理，提高公路通畅能力。实施引洮供水调蓄水池、引洮二期及配套工程，完善田间渠系，提高引洮水资源利用率。加大输气管道规划建设力度，建成兰州至临洮输气管线，新建3条支线输气管道。加快推进电信网、广播电视网、互联网“三网融合”，实现城乡宽带网络全覆盖。

　　（四）加强生态文明建设，全面建成山川秀美的小康社会。围绕“天蓝、地绿、水清”目标，深入实施生态立市战略。以国家生态文明先行示范区、黄土高原生态屏障建设示范区、国家生态屏障建设保护与补偿试验区建设为统揽，实施新一轮退耕还林、天然林保护、三北防护林、飞播造林等重点林业生态工程300万亩，广泛开展全民义务植树活动，推进城镇面山、通道面山和农村“四旁”绿化，每年植树2000万株以上，森林覆盖率达到15%。推进国家水土保持重点工程建设，新修梯田100万亩，新增水土流失综合治理面积1750平方公里、治理程度提高到63%以上。加强大气、水、土壤污染防治，落实生态功能区产业准入负面清单制度，全面推行环境保护督察和“3+”环境监管模式，确保城区空气质量持续改善，城乡饮用水水质达标率达到100%。实施主要河段及重点沟道防洪治理工程，加大地质灾害治理力度，完善自然灾害监测预警系统，提高防灾减灾能力。实施农村人居环境改善工程，80%以上行政村达到生态文明小康村标准。推动循环经济发展，完成通渭国家循环经济示范县、陇西经济开发区循环化改造试点建设，加快定西、临洮和岷县开发区循环化改造。健全垃圾清运和再生资源回收利用体系，城市污水、生活垃圾无害化处理率和废旧农膜回收利用率分别达到90%、95%和80%以上。积极倡导绿色生活方式，开展节约型机关、绿色学校创建等活动，引导全社会共建绿色家园。

　　（五）实施创新驱动战略，全面建成充满活力的小康社会。鼓励企业瞄准优势特色产业转型升级瓶颈问题和战略性新兴产业前沿领域开展联合攻关，争取每年实施重大技改项目100个。加大科技成果转化力度，每年转化科技成果20项，科技进步贡献率达到50%以上。深入推进大众创业、万众创新，开展陇原“双创”五大专项行动，打造众创、众包、众扶、众筹平台，加快建设各类创业创新示范园，每年新增科技企业孵化器、众创空间15 家以上。加快推进国家级和省级试点改革，力争在农村土地确权颁证、综合执法等方面创出更多经验。从“放管服”、环境保护、城市管理、投融资体制等重点领域入手，找准改革切入点，加快破除体制机制障碍，激发经济社会发展活力。稳妥推进农村土地所有权、承包权和经营权“三权分置”，优化土地资源配置，促进适度规模经营发展。积极引进域外金融机构落户定西，优化农村金融服务，提高农业保险覆盖面，有效防范金融风险。强化对外宣传推介，创新节会举办形式，不断扩大中国·定西马铃薯大会暨中国薯都国际论坛、中国中医药产业博览会、陇西李氏文化旅游节、通渭国际书画文化节四大节会影响力，打造全国知名的定西文化节会品牌。持续开展“千亿元大招商”活动，确保招商引资到位资金年均增长10%以上。加快建设“丝绸之路经济带”重要节点城市，按照省上统一部署，积极主动与俄罗斯喀山市缔结友好关系，引导支持市内企业与丝绸之路沿线国家和地区加强交流合作，大力发展外向型经济，不断提高对外开放水平。

　　（六）坚持以人民为中心，全面建成文明和谐的小康社会。积极促进创业，稳定扩大就业，每年新增城镇就业2.6万人以上。瞄准技能人才、新型职业农民、科技人员等七类重点群体，实施有针对性的激励计划，带动城乡居民实现总体增收。坚持教育优先发展战略，基本普及学前教育，推动义务教育均衡发展，全面普及高中阶段教育，大力发展特色职业教育，支持发展高等教育。加快建设健康定西，全面推行健康促进模式，实施中医药传承创新工程，完善医疗服务体系，重视医药卫生人才培养引进，更好满足人民群众的健康需求。落实城乡居民养老保险、低保和临时救助政策，完善失业、工伤、生育保险制度，鼓励农民工参加工伤保险，不断提高社会保障水平。盘活各类闲置资源，多形式、多渠道发展健康养老业，促进养老服务与医疗、健身、家政、保险、旅游等互动发展。改造提升各县区“四馆”和乡镇综合文化站，实施农村广播电视数字化、农家书屋等公共服务改造工程。积极发展健身休闲产业，推动全民健身与全民健康深度融合，加快建设市级体育“两馆两场一中心”和县区“一馆两场”，行政村全民健身设施实现全覆盖。落实粮食安全责任制，提高粮食安全保障能力。深化“文明定西、人人行动”主题活动，深入开展公民素质提升十大行动。坚持依法治市，推进“七五”法制宣传教育工作，完善公共法律服务体系。强化食品药品安全监管，严格落实安全生产责任。创新社会治理方式，积极化解矛盾纠纷，严厉打击各类违法犯罪，维护社会和谐稳定。

2017年重点工作

　　2017年是新一届政府履职的开局之年。全市经济社会发展的主要预期目标是：生产总值增长8%以上，固定资产投资增长12%，规模以上工业增加值增长10%，社会消费品零售总额增长10%，大口径财政收入和一般公共预算收入增长8%，城乡居民人均可支配收入分别增长8%和12%，城镇登记失业率控制在4%以内，居民消费价格总水平控制在3%以内，单位生产总值能耗和污染物排放完成控制指标。

实现上述目标，重点要抓好以下九项工作：

　　（一）全力抓好脱贫攻坚。强化“一号工程”意识，靠实脱贫攻坚责任，减少贫困人口15万人。严格落实“七个一批”脱贫措施，精准分类核实脱贫人数，建立到户工作台账，确保贫困人口按需求得到帮扶。重点培育发展农民专业合作社等新型经营主体，完善利益联结机制，引导贫困户至少参加1个农民专业合作经济组织。加快推进7县区光伏扶贫试点建设，带动发展贫困户1.6万户。加大项目和资金争取力度，改造农村危房1.09万户。实施易地扶贫搬迁项目41个，搬迁4.7万人。建设贫困村幼儿园291个、综合性文化服务中心134个、“一村一场”（篮球场）200个。精准培训贫困劳动力6.5万人，保证贫困家庭至少有1人取得职业资格证书、掌握一门致富技能。实施千企帮千村精准扶贫行动，广泛动员社会力量加大帮扶力度。

　　（二）强力推进项目建设。认真研究对接国家和省上重大战略、重大政策，筹措项目前期费6000万元，争取到位国家专项资金60亿元以上。实施500万元以上项目1800个，力争固定资产投资突破700亿元。积极争取国家专项建设基金，实施政府与社会资本合作项目，撬动民间投资330亿元以上。争取3条地方高速公路和陇西至渭源等3条支线输气管道开工建设。全力配合省属重点项目建设，完成宝兰客专定西西站、通渭站前广场和兰渝铁路渭源、漳县、岷县站前广场建设任务，加快引洮二期、渭武高速建设进度。加大招商引资力度，强化签约项目跟踪服务，争取招商引资到位资金460亿元以上，同比增长10%。

　　（三）大力发展现代农业。推动良种良法配套、农机农艺融合，加快发展旱作高效农业、设施观光农业、生态循环农业，完成全膜双垄沟播种植364万亩，粮食总产量稳定在150万吨以上。积极推进安定、临洮、陇西渭北3个省级以上现代农业示范区建设，扶持壮大54户省级以上农业产业化龙头企业，不断提高农业生产经营组织化、规模化、标准化、机械化水平。加强道地中药材优良品种选育推广，建立标准化种植基地80万亩，金银花示范面积扩大到3.5万亩，筹建定西中药材商品交易中心，加快国家中医药产业发展综合试验区建设。深入实施马铃薯主食产业开发行动，引进扩繁主食化专用品种2个以上，建立原种扩繁基地6万亩、主食化品种基地130万亩，争取实施主食产品精深加工项目10个，加工能力达到13万吨。种植优质牧草150万亩，扶持发展牧草加工龙头企业12家，新发展规模养殖场150个、家庭养殖场1000个、规模养殖户1万户，畜禽饲养总量达到3100万头只。种植蔬菜80万亩，发展设施农业2万亩，新建林果基地10万亩。

　　（四）加快发展工业经济。加快推进工业领域供给侧结构性改革，推动传统产业改造升级，培育壮大新型工业，完成工业增加值38.3亿元，同比增长9.5%。实施千万元以上重点工业项目135个，完成投资125亿元，薯香园马铃薯主食产品产业化开发等26个项目建成投产，江能医药科技产业园、岷海制药当归生产等60个项目完成年度建设任务。积极开拓市场，扩大外销总量，促进有色冶金、新型建材、特色农产品实现产销平衡，推动工业领域去库存。完成定西高强度螺钉公司核电产品专用紧固件生产基地建设、圣大方舟环保生物质基材生产等35个项目技改任务，提高企业自主创新能力。新建省级企业技术中心、工程技术研究中心和重点实验室5个，培育认定高新技术企业2家。支持扶正药业、圣大方舟、凯凯农科等企业通过资本市场挂牌融资或发行债券，新增规模以上工业企业15户。加快定西经济开发区循环经济园二期河西片、商住商服区道路工程建设，经济开发区和工业集中区完成投资150亿元，新引进企业50户以上。

　　（五）培育壮大新兴产业。实施陇中文化旅游创意产业园等36个文化旅游项目，加快推进贵清山/遮阳山、渭河源、狼渡滩等景区房车露营基地建设，实现文化产业增加值7.38亿元、旅游综合收入30亿元。积极创造条件，支持陇西一方、普尔康、岷海制药等企业开展中药配方颗粒生产。加强与中国农科院兰州牧研所等院校合作，开展动物血清、兽药粉预混剂等新产品研发生产。加快推进定西军民融合（西北）应急物流基地和定西铁路物流中心等大型物流项目前期工作，完成亿联国际商贸城二期、临洮南部农副产品集散中心等项目年度建设任务，金帆现代物流产业园建成运营。深入推进渭源县全国电子商务进农村综合示范县项目建设，岷县全国电子商务进农村综合示范县项目通过验收，70%以上的行政村建成电子商务服务站点，交易额增长20%。

　　（六）统筹推进城乡建设。完成城镇风貌规划编制，启动通渭、渭源县城新一轮总体规划修编，村庄规划编制覆盖率达到90%以上。市区实施城建项目100个，完成投资87亿元以上。启动实施市博览中心陈列布展、市体育场等公共服务设施项目，加快推进集中供热管网改造、城市道路维修及绿化、西川停车场等基础设施建设项目，亚行贷款城市道路、安定广场等项目建成投入使用，打通中华路南段丁字路，在市政府旧址规划建设市区中心广场。各县城区和重点小城镇实施项目165个，完成投资97亿元以上。加快推进通渭平襄书画小镇等3个省级特色小镇建设，全面启动市级特色小镇创建工作，积极申报国家级特色小镇。以国省道沿线为重点，开展农村环境综合整治，建成美丽示范村40个、环境整洁村150个。重点治理小区物业、广告牌匾、占道经营、餐饮油烟、交通秩序、违章建筑、流浪动物等突出问题，不断加强和改进城市管理。启动实施10条省道提升改造工程，完成定西至渭源等8条省道和二级公路年度建设任务，加快推进定西综合客运中心及南北客运站建设。优化引洮一期配套工程，开工建设内官、马河、王家河引洮供水调蓄工程，关闭引洮受益区地下水井。

　　（七）着力加强生态建设。实施重点林业生态工程，完成造林封育54万亩。深入开展全民义务植树，整区域推进造林绿化和“四旁”绿化，完成植树2000万株。加强森林资源管护，巩固造林绿化成果。实施国家水土保持和淤地坝除险加固等项目，新修梯田20万亩，新增水土流失综合治理面积350平方公里。开展第二次全国污染源普查，新增空气质量监测点8个。实施渭河流域综合整治及饮用水水源地保护等项目，确保城乡供水水质达标安全。加快污水、垃圾处理设施建设，积极实施垃圾发电项目，新增污水日处理量750吨、垃圾日处理量100吨。严格落实节能减排目标责任制，加快推进定西、临洮、岷县省级开发区循环化改造，开展特色农产品加工和传统制造业绿色改造。为所有乡镇和行政村灾害信息员配备应急救灾设备，全面提升基层防灾减灾救灾能力。

　　（八）全面推进各项改革。深化全民参保登记计划、陇西县农村集体经营性建设用地入市等全国改革试点，综合执法、中药材产值保险等全省改革试点，力争形成经验并积极推广。推进投融资体制改革，推广政府和社会资本合作模式，支持社会资本进入特许经营领域建设运营。开展水、电、能源等领域价格改革，全面放开竞争性领域商品和服务价格。完成21个国有林场改革任务。启动城市公立医院综合改革，加快推进分级诊疗制度建设，基层医疗卫生机构诊疗量占总诊疗的65%以上，县域内就诊率提高到90%。设立产业发展基金，探索开展蔬菜价格和牛羊养殖保险，推广中药材产值保险、互助资金担保增信等更多金融创新产品。

　　（九）协调发展社会事业。认真落实扶持创业、促进就业政策，新增城镇就业2.7万人。调整优化教育布局，完成城区学校教育资源整合。新建幼儿园555个，1000人以上行政村幼儿园实现全覆盖。提高教育教学质量，高考二本以上上线率达到35%以上。创新职业教育办学模式，支持民办教育规范发展。实施重点卫生项目18个，市医院住院楼及儿童病区等11个项目建成投用，市妇幼保健院和疾控中心业务用房等7个项目完成主体工程，医疗保险异地结算平台建成并网运行。积极创建新一轮全国计划生育优质服务先进单位。提高城乡低保和农村五保保障标准，健全完善城乡医疗救助制度，医前、医中救助比例达到20%以上。大力推进科技特派员到基层创新创业，争创全省科技特派员示范市。完成中央地面数字广播电视覆盖工程，广泛应用新媒体，拓宽广播电视节目传播渠道。推进食品药品生产经营使用规范化建设，保障群众饮食、用药安全。认真落实安全生产“一岗双责”，坚决遏制重特大事故发生。

　　各位代表！2017年，市政府将重点聚焦脱贫攻坚，继续为全市人民办好十件实事：1.为贫困村配套互助资金4345万元，每村市级配套资金达到10万元。2.改造提升农村供水工程，扩大供水管网覆盖和入户范围，农村自来水普及率达到91%以上。3.新建撤并建制村通畅工程380公里、县乡道路安全生命防护工程284公里。4.为326所学校改造建设校舍16.2万平方米，培训校园安全管理人员600名。5.实施10个乡镇卫生院基层中医药服务能力建设项目，培训贫困村村医400名，为1.7万对计划内怀孕的农村育龄夫妇开展免费孕前优生健康检查。6.市县区财政补贴900万元，帮助20万“两保一孤”特困人群办理意外伤害附加重大疾病保险。7.提升改造城区面山绿化3.1万亩。8.实施棚户区改造4100户。9.扶持200名普通高校毕业生到市内企业就业。10.为困难群众办理法律援助案件2750件。

努力建设人民满意的政府

　　繁重而艰巨的发展任务，对政府自身建设提出了新的更高要求。我们要坚持依宪施政、依法行政、简政放权，切实转变政府职能，把全面建成小康社会使命扛在肩上，把万家忧乐放在心头，增强政府执行力和公信力，努力建设人民满意的政府。

　　（一）坚持依法行政，建设法治型政府。坚持把依法行政贯穿政府工作全过程，做到法定职责必须为、法无授权不可为。认真落实政府常务会议会前学法制度，完善集中学习培训制度，提高各级干部法治思维和依法行政水平。进一步健全重大行政决策制度和政府法律顾问制度，建立公职律师制度，推动决策科学化、民主化、法制化。巩固提升综合执法改革试点成效，建立权责统一、权威高效的行政执法体系。认真执行市人大及其常委会的决议决定，自觉接受人大依法监督和政协民主监督。积极配合市人大常委会地方立法工作，探索制定政府规章。及时办理人大代表意见建议和政协委员提案，不断提高办理质量。

　　（二）坚持求真务实，建设责任型政府。深入践行“三严三实”和“四讲四有”合格党员要求，增强政治意识、大局意识、核心意识、看齐意识。推进政府管理创新，健全并严格执行工作责任制，确保重大政策措施和工作任务不折不扣落到实处。全面贯彻《中国共产党党内监督条例》，健全督查问责机制，加强政务督查和效能监督，完善常态化督查工作机制，坚决整治庸懒散慢拖和不作为、乱作为等问题，做到有责必问、有错必究。健全正向激励机制，对愿干事、敢干事、能干事的干部加大支持和奖励力度，为改革创新者撑腰鼓劲。

　　（三）坚持履职为民，建设服务型政府。始终把人民的期盼作为奋斗目标，强化宗旨意识，密切联系群众，坚持问政于民、问需于民、问计于民，及时研究解决群众关心的热点难点问题。持续推进简政放权，大力推广“互联网+政务服务”，对行政权力事项和责任事项实行清单化、标准化、动态化管理，推进网上“晒权”“行权”“监权”，最大限度利企便民，营造大众创业、万众创新良好环境。强化事中事后监管，全面推行“双随机、一公开”机制，降低监管成本，提高监管效率。推进政府职责优化配置和统筹整合，减少部门职能交叉，理顺职责关系，确保政府工作顺畅高效运转。

　　（四）坚持公开透明，建设阳光型政府。大力推行政务公开，突出抓好财政预决算、公共资源配置、重大项目建设等信息公开，做到行政决策公开、执行公开、管理公开、服务公开和结果公开，主动接受社会和舆论监督。推进政务信息服务平台建设，完善例行发布、应急发布、政策解读等机制，着力提高政务运行质量和水平。加强政府门户网站建设，不断提升传播能力，着力打造互联网时代政府信息公开平台、便民服务平台、政民互动平台。充分发挥新兴媒体和网络平台作用，建立健全政务舆情收集、研判、处置和回应机制，加强互动交流，及时回应社会关切。

　　（五）坚持勤政廉政，建设廉洁型政府。认真落实从严治党要求，严格执行《关于新形势下党内政治生活的若干准则》，切实加强政府系统思想政治建设，深入推进机关党建和党风廉政建设，为推动改革发展提供坚强保障。严格落实“一岗双责”，坚持廉政建设与业务工作两手抓、两手硬，发挥行政监察、审计监督作用，突出重点领域、关键环节和重要岗位的监督管理，推进审计全覆盖，切实规范行政权力运行。严格执行中央“八项规定”、国务院“约法三章”和省委、市委相关规定，厉行勤俭节约，反对铺张浪费，持续压缩“三公”经费，进一步提高财政资金使用效益。坚决纠正侵害群众利益的不正之风，坚定不移惩治腐败，着力营造风清气正、崇廉尚俭、遵纪守法的干事创业氛围。

　　各位代表！蓝图已绘就，奋进正当时。让我们更加紧密地团结在以习近平同志为核心的党中央周围，在省委、省政府和市委的坚强领导下，紧紧围绕市第四次党代会的战略部署，凝心聚力，开拓进取，奋力拼搏，坚决打赢脱贫攻坚战，为与全省全国同步全面建成小康社会、建设幸福美好新定西而努力奋斗!
